# An Insight on Novel Molecular Pathways in Metastatic Prostate Cancer: A Focus on DDR, MSI and AKT

**DOI:** 10.3390/ijms222413519

**Published:** 2021-12-16

**Authors:** Veronica Mollica, Andrea Marchetti, Matteo Rosellini, Giacomo Nuvola, Alessandro Rizzo, Matteo Santoni, Alessia Cimadamore, Rodolfo Montironi, Francesco Massari

**Affiliations:** 1 Medical Oncology, IRCCS Azienda Ospedaliero-Universitaria di Bologna, Via Albertoni-15, 40138 Bologna, Italy; veronica.mollica7@gmail.com (V.M.); andrea.marchetti12@studio.unibo.it (A.M.); matteo.rosellini@studio.unibo.it (M.R.); giacomo.nuvola87@gmail.com (G.N.); rizzo.alessandro179@gmail.com (A.R.); fmassari79@gmail.com (F.M.); 2 Oncology Unit, Macerata Hospital, 62100 Macerata, Italy; mattymo@alice.it; 3 Section of Pathological Anatomy, School of Medicine, Polytechnic University of the Marche Region, United Hospitals, 60126 Ancona, Italy; 4 Molecular Medicine and Cell Therapy Foundation, Department of Clinical and Molecular Sciences, Polytechnic University of the Marche Region, 60100 Ancona, Italy; r.montironi@staff.univpm.it

**Keywords:** AKT, DDR, immunotherapy, mCRPC, MSI, PARP inhibitors, PI3K, prostate cancer

## Abstract

Prostate cancer is still one of the main causes of cancer-related death in the male population, regardless of the advancements in the treatment scenario. The genetic knowledge on prostate cancer is widely increasing, allowing researchers to identify novel promising molecular targets and treatment approaches. Genomic profiling has evidenced that DNA damage repair genes’ alterations are quite frequent in metastatic, castration resistant prostate cancer and specific therapies can interfere with this pathway, showing promising activity in this setting. Microsatellite instability is gaining attention as it seems to represent a predictive factor of the response to immunotherapy. Furthermore, the PTEN-PI3K-AKT pathway is another possible treatment target being investigated. In this review, we explore the current knowledge on these frequent genomic alterations of metastatic prostate cancer, their possible therapeutic repercussions and the promising future treatments under evaluation.

## 1. Introduction

**Figure 1:**
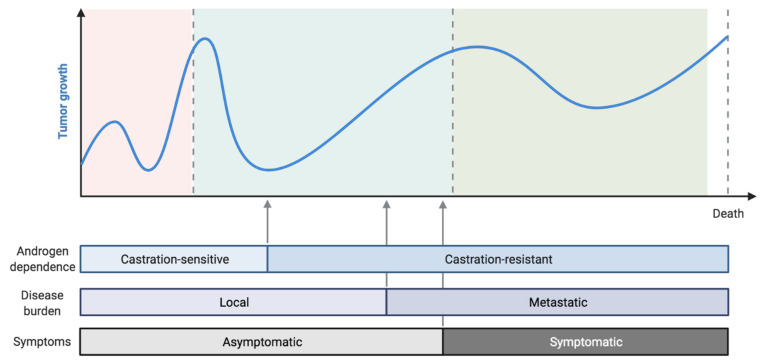
Schematic figure representing the natural history of prostate cancer.

Prostate cancer (PCa) is still the most frequent type of malignant neoplasia in men and represents a leading cancer-related cause of death [1]. PCa cells’ proliferation is highly dependent on hormonal stimulation driven by androgens. Therefore, androgen-deprivation therapy (ADT) is the treatment cornerstone, associated with improved clinical outcomes in both the hormone sensitive and castration resistant setting [2]. Unfortunately, after a variable period of time, hormone sensitive prostate cancer (HSPC) cells will eventually develop resistance to ADT, leading to a castration resistant prostate cancer (CRPC) scenario (Figure 1). In this setting, approved treatments among the different stages of the disease are taxane chemotherapy (docetaxel and cabazitaxel), androgen receptor targeting agents (ARTAs), such as enzalutamide and abiraterone acetate, and, recently, the Poly ADP-ribose polymerase (PARP) inhibitor olaparib [3,4,5,6,7,8,9].

However, the majority of patients will eventually develop resistance to treatments. Thus, the research and identification of further hormonal pathway modifications and other genomic alterations leading to cancer progression and resistance to therapies is of pivotal importance to individuate new possible therapeutic strategies [10,11]. The knowledge on molecular alterations driving prostate cancer progression and development or resistance to standard treatment, and that could have prognostic implications is exponentially growing, thus allowing researchers to find new therapeutic targets and improve patients’ care. Multiple genomic studies based on whole-exome sequencing or transcriptome data allowed for the identification of several molecular pathways altered in advanced PCa: not only the most frequent and explored androgen receptor signaling pathway, present in over 60% of metastatic CRPC (mCRPC) cases, but also *ETS*, *TP53*, DNA damage repair (DDR) genes, phosphatase and tensin homolog (*PTEN*)-phosphatidylinositol 3-kinase (*PI3K*)-*AKT* signaling, and mismatch repair genes [12,13]. Consequently, novel therapeutic targets are under investigation and, in particular, the promising fields are the DDR genes alterations, microsatellite instability (MSI), and the PTEN-PI3K-AKT pathway.

In this review of the literature, we will explore and present recent data on the DDR, MSI and PTEN-AKT pathways and the future possible therapeutic implications of these molecular alterations.

## 2. DDR Alterations, PARP Inhibitors and Platinum-Based Chemotherapy

### 2.1. DNA Damage Response Systems 

DDR defects are common in various types of cancer and are also observed in about 20% of mCRPC, in which the most frequent mutations are localized in the homologous recombination repair (HRR) genes, like *ATM*, *BRCA1* and *BRCA2* [14]. 

As emerged from a multicenter study, the incidence of germline mutations in DDR genes among men with mCRPC is near to 12%, not differing in a consistent way according to age or family history of PCa [15]. On the contrary, these alterations are less represented in men with localized disease. Among the DDR genes, the most frequent germline mutation in mCRPC is *BRCA2*, as evidenced in a multicenter study conducted by the Stand Up to Cancer–Prostate Cancer Foundation (SU2C–PCF) consortium [15,16]. Furthermore, in this trial 8% of the mCRPC presented germline mutations in DDR genes, while 23% harbored somatic alterations [12].

**Figure 2:**
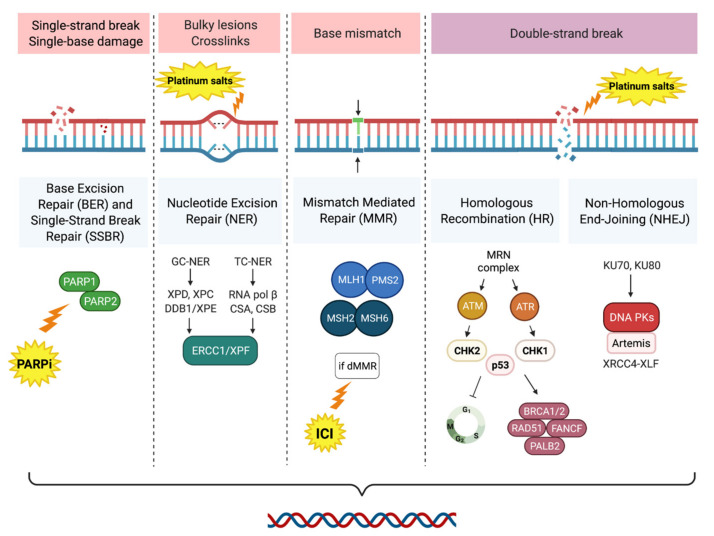
Schematic illustration of DDR pathways. Platinum compounds provoke intrastrand adducts and interstrand DNA crosslinks that can be repaired only by the activation of the NER, HR and NEHJ systems. PARPi inhibits the single-strand break repair mediated by BER and SSBR. Deficit in the mismatch repair leads to the increase of mutations and, consequently, neoantigens. This process is associated with a potential response to ICI. dMMR: deficit mismatch repair; HR homologous recombination; ICI: immune checkpoint inhibitor; NER: nucleotide excision repair; NEHJ: non-homologous end joining; PARPi: PARP inhibitor; SSBR, single-strand break repair.

Different DNA repair mechanisms can be activated by the cell in order to prevent its genomic integrity. These mechanisms have complementary and in part overlapping pathways. When a dysfunction occurs in these systems, the human cell is not able to restore the genomic damages that may constantly take place, leading to an instability in the genome which enhances the carcinogenesis’ process. Both DNA strands could be damaged and, in this scenario, the main systems involved in the repairing process are non-homologous end joining (NHEJ) and homologous recombination (HR). HR requires several mediators such as *BRCA1*, *BRCA2*, *PALB2*, *CHECK2*, *RAD51*, and *ATM*. On the other hand, if the genomic insult occurs in only one strand, the unaltered one is employed as a framework by mismatch repair (MMR), nucleotide excision repair (NER), base excision repair (BER), and single-strand break repair (SSBR) [16] (Figure 2).

**Figure 3:**
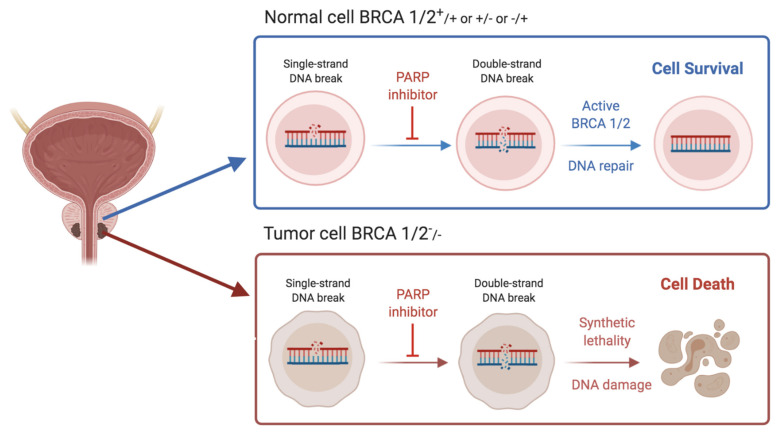
Figure schematically representing the mechanisms of action of PARP inhibitors treatment for patients with BRCA-mutant prostate cancer. BRCA1 and BRCA2 genes have been suggested to encode proteins of pivotal importance to DNA homologous recombination repair processes.

*PARP1* and *PARP2*, proteins that participate in the fixing of single-strand breaks, are blocked by PARP inhibitors (PARPi). The antitumoral effect granted by this class of drugs is displayed in case of lacking *BRCA1/2* genes, because consequently the HRR mechanism is not able to repair the damages induced by the PARPi, thus causing cell death [17] (Figure 3).

### 2.2. Main Trials on PARPi in Metastatic Prostate Cancer and New Approaches under Investigation

The TOPARP phase II two-stage trial was designed to evaluate the efficacy of the PARPi olaparib in pretreated mCRPC patients. The first of the two studies, TOPARP-A, showed the antitumoral activity of olaparib 400 mg BID in 16 patients evaluable for response with mCRPC previously treated with chemotherapy and novel anti-androgens (all had received docetaxel, 58% also cabazitaxel and 98% received abiraterone or enzalutamide), and with DDR genes defects [18]. Fourteen of these sixteen patients (88%) presented a response to olaparib, in particular all seven patients with *BRCA2* loss and four of the five patients with *ATM* aberrations. About 22% of patients had a reduction of PSA levels of at least 50%, and 29% of circulating tumor-cell count to less than five cells/7.5 mL. 

In 2019, the second trial, TOPARP-B, confirmed the efficacy of olaparib in men with mCRPC and DDR aberrations, who had received one or two previous taxane lines of therapy [19]. Confirmed response was the primary endpoint, achieved by 54.3% of patients who had received olaparib 400 mg BID and by 39.1% of patients who had received 300 mg BID.

The recently published phase III PROfound study tested olaparib 300 mg BID in mCRPC patients who experienced progression of the disease during the treatment with a new hormonal agent, enzalutamide or abiraterone (previous taxane chemotherapy was permitted) [9]. In cohort A, in which patients had an alteration in *BRCA1*, *BRCA2* or *ATM*, the primary endpoint of imaging-based progression-free survival (PFS) was longer in the olaparib group than in the control group that received enzalutamide or abiraterone (median, 7.4 months versus 3.6 months, hazard ratio (HR) 0.34). In cohort B, men had aberrations in 12 other genes involved in the DDR system [9]. Moreover, the overall survival (OS) final analysis demonstrated a consistent benefit for olaparib in both cohorts (cohort A, 19.1 months versus 14.7 months, HR 0.69; cohort B, 14.1 months versus 11.5 months) [20].

An open-label phase II trial, TALAPRO-1, investigated another PARPi, talazoparib, in 127 mCRPC patients progressed to enzalutamide or abiraterone, who had received at least one taxane therapy for metastatic disease [21]. The overall response rate (ORR) was 29.8% and the most common grade three or four adverse events were anemia (31%), thrombocytopenia (9%) and neutropenia (8%). On the basis of this promising result, a TALAPRO-2 (NCT03395197) phase III trial was designed, evaluating talazoparib plus enzalutamide as a first line therapy in patients affected by mCRPC, with or without HRR gene defects. This trial is still ongoing. In addition, another phase III trial, TALAPRO-3 (NCT04821622), is still in the recruiting stage and is going to investigate talazoparib plus enzalutamide in a HSPC setting in men with HRR aberrations.

As for niraparib, the phase II single-arm GALAHAD trial enrolled patients with mCRPC with DDR alterations (and resistance to androgen receptor-target treatment and taxanes) to receive niraparib. Preliminary results showed an ORR of 41% vs. 9%, in the BRCA-mutated population and in patients with BRCA wild type, respectively, with a median rPFS of 8.2 months and a median OS of 12.6 months [22]. This PARPi is currently under investigation in another study, the phase III double-blind MAGNITUDE (NCT03748641), in which it is associated with abiraterone in untreated mCRPC patients and compared to placebo + abiraterone (the population will be stratified on the base of DDR-genes status).

Furthermore, the PARPi rucaparib showed some antitumor activity in the TRITON2 study in patients with mCRPC and germline or somatic BRCA mutations, consisting in an ORR of 50.8% in patients with measurable disease [23] 

All these studies mentioned above are summarized in Table 1.

The combination of PARPi with chemotherapy seems to be a promising strategy, due to their presumable synergic mechanism, but a study that evaluated veliparib plus temozolomide did not show great activity of this combination treatment approach [24]. Similarly, veliparib plus abiraterone in mCRPC did not improve outcomes compared to abiraterone alone [25]. 

An interesting approach that has been explored in preclinical analysis is to combine histone deacetylase inhibitors (HDACi) and PARPi, in order to increase the DNA-damage and to maximize the death of prostate cancer cells [26]. 

Furthermore, inhibitors of other proteins involved in the DDR mechanism are currently under investigation. The TRAP trial (NCT03787680) is evaluating the ATR inhibitor AZD6738 in combination with olaparib in mCRPC, in both DDR proficient and deficient patients. The Wee1 kinase inhibitor AZD1775 is currently being analyzed in advanced solid tumors in a phase I trial (NCT01748825) and the Chk1/2 Inhibitor (LY2606368). The Chk1/2 Inhibitor (LY2606368) is another approach explored in a phase II single arm trial in mCRPC, breast and ovarian cancer (NCT02203513).

Lastly, other agents have been tested in preclinical studies combined with PARPi, such as CDK1 inhibitors [27], PI3K or AKT inhibitors, CDK12 inhibitors and HSP90 inhibitors. The aim of these combinations is to make HRR proficient tumors’ cells sensitive to platinum chemotherapy and PARPi [28,29].

### 2.3. Role of Platinum-Based Chemotherapy

Nowadays, platinum chemotherapy (P-CHT) in unselected mCRPC patients is not a standard in clinical practice. As a matter of fact, every study that has investigated this strategy failed in demonstrating a survival benefit, including the phase III SPARC trial in which oral satraplatin plus prednisone improved pain and delayed the progression of disease in mCRPC, but did not improve the OS compared to a placebo plus prednisone (all men had received one previous line of chemotherapy) [30]. Interestingly, Mota et al. demonstrated a better and longer response to the platinum compounds, along with more frequent 50% PSA-decrease from the baseline in men with mCRPC with DDR genes mutations compared to patients without alterations [31]. No survival benefit was highlighted between the two populations. Moreover, BRCA-mutated patients responded to the platinum chemotherapy even after progression to PARPi [32]. This better response has a strong biological rationale. Indeed, platinum agents create adducts in DNA that impede replication and transcription, producing a consequent damage not repairable by the altered DDR system [33].

In addition, platinum agents play a pivotal role in the treatment of prostate cancer that transitions to small-cell carcinoma or neuroendocrine tumors, thus acquiring resistance to hormone therapy and loss of the androgen receptor signaling pathway [34]. A prospective study evaluated 148 metastatic tumor biopsies of patients with progressive disease after ARTAs, revealing histological neuroendocrine features in about 17% of the samples [34]. Several molecular biomarkers are currently under investigation, such as the combined loss of *TP53* and *RB1*, in order to identify early and select those patients that have undergone this process of lineage plasticity, that is associated with a worse OS [35].

### 2.4. PARPi and Platinum Salts: Current and Future Directions

In conclusion, PARPi are effective and available treatment weapons in DDR genes mutated patients in the mCRPC setting, mainly on the basis of the PROfound and TRITON2 trials. On the other hand, P-CHT has a promising role in these selected patients but holds a more valuable activity in the case of neuroendocrine differentiation. 

In consideration of the quite high frequency of germline mutation in DDR genes in mCRPC, a genetic test is now recommended by the National Comprehensive Cancer Network guidelines in all patients in this setting [23].

With regards to the screening phase, in 2019 Page et al. published the interim results of the IMPACT study that showed the association between *BRCA2* mutation and both a higher incidence of prostate cancer and a younger age at diagnosis, suggesting the utility of PSA screening in men with this genetic alteration [36].

In all this promising and evolving context, there are various questions that must be answered. First of all, there is no precise indication of which DDR genes should be included in the next-generation sequencing (NGS) panel (e.g., FoundationOne CDx was used in PROfound while GeneRead DNAseq was used in TOPARP-A) or the right moment in the course of the disease when this test should be performed. Finally, it is still unclear what the best timing is or the right sequence of treatment between PARPi and P-CHT [37].

## 3. Microsatellite Instability and Immunotherapy

### 3.1. Microsatellite Instability as a Predictor for Immunotherapy

In the last few years, immunotherapy has become one of the most important cancer treatment modalities, drastically modifying the natural history and prognosis of several malignancies previously characterized by a ruinous and rapid evolution (i.e., renal cell carcinoma, melanoma or non-small cell lung cancer). Particularly, the immune checkpoint blockade has revolutionized the field of tumor immunotherapy, since the approval of ipilimumab for advanced melanoma in 2011 [38]. Despite the meaningful benefits derived from this strategy, immunotherapy is not effective in all patients, thus explaining the physicians’ constant search for predictive markers to select responders.

The term “microsatellite instability” suggests a molecular tumor phenotype resulting from genomic hypermutability, whose main feature is the gain or loss of nucleotides from microsatellite tracts, consisting of DNA elements composed of short repeating motifs [36,39]. This genomic hypermutability is the consequence of the loss of function of many MMR genes, including the MutL homologue 1 (*MLH1*), post-meiotic segregation increased 2 (*PMS2*), MutS homologue 2 (*MSH2*), and MutS 6 (*MSH6*) genes. Therefore, microsatellite instability-high (MSI-H) malignant cells are unable to successfully correct mistakes that have occurred during DNA replication. Defects in MMR proteins and the subsequent MSI-H state led to the collection of mutational loads in cancer-related genes along with several neoantigens’ synthesis, thus enhancing the anti-tumor immune response of the host [40,41]. The loss of function in the MMR genes or the related MSI-H status define a subset of patients as high responders to immune checkpoint blockade, therefore MSI-H is now considered a predictive factor for immunotherapy [41]. All these observations have provided the biological rationale to investigate immune checkpoint inhibitors (ICIs) as a therapeutic strategy in deficient MMR (dMMR)/MSI-H solid tumors, paving the way for the phase II KEYNOTE-158 trial in which the clinical benefit of the anti-programmed death-1 (PD-1) pembrolizumab was shown in 27 types of previously treated and metastatic dMMR/MSI-H non-colorectal malignancies [42]. As a result, in 2017 the US Food and Drug Administration (FDA) granted the first historical tissue/site-agnostic approval of pembrolizumab for unresectable or advanced dMMR/MSI-H cancers, that have progressed after a previous standard therapy and in the absence of satisfactory alternative therapeutic options, regardless of the primary tumor’s histology [43]. Nevertheless, some solid tumors have demonstrated lower response rates, such as pancreatic and central nervous system malignancies, challenging the use of dMMR/MSI-H as an unfailing agnostic biomarker [44].

### 3.2. Microsatellite Instability and Prostate Cancer

Focusing on prostate cancer, several studies highlighted the immune-exclusive quality of its microenvironment due to a large variety of features, including a low tumor mutational burden with a decreased neoantigen expression, hyperactivity of myeloid-derived suppressor cells (MDSCs) and regulatory T-cells, loss of major histocompatibility complex (MHC) class I expression and abnormal IFN-1 signaling [45]. The frequency of dMMR/MSI-H varies between 1% and 12% in mCRPC patients, with an unclear prevalence [46], but it should be pointed out that different assays used to detect dMMR tumors may lead to an important discordance [47]. In a study of 60 rapid tumor autopsies from metastatic PCa patients, 12% (*n* = 7) were described as hypermutated, being dMMR as well as MSI-H [48]. Furthermore, MSI-H frequency was found to be 3% in another study of tumor biopsies from 150 mCRPC patients [12].

Like other solid tumors, it has been shown that dMMR or MSI-H prostate cancer may respond better to the immune checkpoint blockade [49]. As outlined in their integrated analysis of transcriptomic, genomic and clinical data, Rodriguez and colleagues displayed higher intra-tumoral T-cell infiltration and immune checkpoint-related transcripts in dMMR PCa. In more detail, among the four mutational signatures described, the dMMR signature (including MSI-H PCa) is associated with an overexpression of MDSCs’ accumulation related genes, such as *JAK2*, *VCAM1* and *NLPR3* [47]. Moreover, 3.1% (*n* = 32) of the 1033 mCRPC patients enrolled in a 2019 single institution experience at the Memorial Sloan Kettering Cancer Center were characterized by MSI-H prostate malignancy, and 21.9% of these latter patients carried a Lynch syndrome-associated germline mutation. An ICI-based therapy was administered in 11 out of these 32 patients, and about half of them gained >50% reduction in PSA value, while four men achieved a radiological response [50]. Recent trials have unveiled a meaningful relationship between the ductal histology (which is aggressive and rare) and DDR genes’ alterations as well as dMMR/MSI-H. A small sample size-based study by Schweizer et al. showed that 4 out of 10 ductal PCa patients were dMMR, and 3 of them were also characterized by MSI-H. Notably, one of these dMMR/MSI-H patients with ductal PCa achieved a significant reduction in PSA levels during treatment with pembrolizumab [51].

Despite all these observations, immunotherapy still has difficulty fitting into daily clinical practice for the treatment of metastatic PCa, although, dMMR and MSI-H may surely be considered as useful biomarkers to identify subsets of PCa patients who are most likely to respond to immunotherapy, along with PD-L1 status, tumor mutational burden (TMB) and other predictive factors. Nonetheless, the overall modest efficacy of ICIs in PCa patients sheds light on the need to improve the predictive ability to detect responders, by also making the most of an integration of different biomarkers [52]. 

### 3.3. Immunotherapy in mCRPC: Vaccines

Based on the phase III IMPACT trial’s results, Sipuleucel-T was the first immunotherapeutic agent to be approved by the US FDA for mCRPC in 2010, as well as the first ever autologous cellular therapeutic vaccine for any tumor [53]. Triggering the immunological T-cell response against tumor-associated antigens represents the basis of the cancer vaccines’ pharmacodynamic. Sipuleucel-T is an autologous cell vaccine prepared from peripheral blood mononuclear cells, extracted during patients’ leukapheresis and incubated with PA2014 (a recombinant fusion protein of GM-CSF and prostatic acid phosphatases), in order to elicit an antitumor immune response [54]. According to the IMPACT trial’s data, the administration of this cancer vaccine led to a significantly increased OS when compared to the placebo (HR 0.78, *p* = 0.003), even if no differences were detected in terms of PFS or PSA decline [53]. Several studies have so far highlighted how limited the use of this vaccine is in everyday clinical practice [55], but the increasing knowledge of other tumoral antigens—such as PSA—can promote many investigations assessing new anti-PCa vaccines [52]. 

### 3.4. Immunotherapy in mCRPC: Anti-CTLA-4 and Anti-PD-1/PD-L1 Agents

Immune checkpoints regulate many pathways that are exploited by cancer cells to escape the anti-tumor immunological response, by inhibiting T-cell activity and supporting the development of an immune suppressive microenvironment. Monoclonal antibodies targeting these immune checkpoints (PD-1, programmed death-ligand1-PD-L1- or cytotoxic T-lymphocyte antigen 4-CTLA-4) have been investigated as mCRPC treatments with mainly discouraging results. First of all, the anti-PD-1 pembrolizumab showed a limited clinical activity in the phase IB KEYNOTE-028 trial, by achieving an ORR of 17.4% (with four partial responses and eight stable disease outcomes) along with humble survival results in previously treated mCRPC patients with PD-L1 expression ≥ 1% [56]. The phase II KEYNOTE-199 study investigated pembrolizumab in mCRPC patients, pretreated with docetaxel and at least one androgen-receptor inhibitor. Pembrolizumab activity was displayed in both PD-L1 positive and negative patients (ORR 6% and 3%, respectively), as well as in men with bone-predominant disease, with a median OS of 9.5 months, 7.9 months and 14.1 months, respectively [57]. In the above-mentioned studies, the safety profile of pembrolizumab was consistent with other tumor types. 

The anti-CTLA-4 ipilimumab was first tested in 14 patients with mCRPC, highlighting a PSA decline of ≥50% in two men and <50% in eight men, along with no objective response according to RECIST criteria [58]. The subsequent randomized phase III CA184-043 and CA184-095 trials evaluated the safety and efficacy of ipilimumab in mCRPC patients progressed after docetaxel and previously treated with bone-directed radiotherapy or untreated asymptomatic/paucisymptomatic patients without visceral metastases. Ipilimumab did not meet its primary endpoint of OS in the CA184-043 study (HR 0.85, *p* = 0.053) nor in the CA184-095 trial (HR 1.11, *p* = 0.3667) [59,60]. Notably, the median PFS was higher with ipilimumab in both studies, and prespecified subgroup analyses unveiled the more significant benefit related to this ICI in highly pretreated patients with favorable prognostic factors, including the absence of visceral metastases [52]. The anti-PD-L1 atezolizumab showed a 12-months OS rate of 52.3% and a 6-months PFS rate of 26.7% when administered in metastatic hormone-refractory patients who had progressed on enzalutamide and/or Sipuleucel-T. Moreover, atezolizumab was well tolerated, and demonstrated evidence of limited disease control (partial response 9%, stable disease 45%) [61]. The expansion cohort of the phase IA JAVELIN Solid Tumor trial tested the monoclonal anti-PD-L1 antibody avelumab in 18 pretreated mCRPC patients with limited results: only seven patients witnessed a >24-months stable disease and only three men showed an increased PSA doubling time (PSADT) [62]. In Table 2 an overview of the ongoing clinical trials evaluating the clinical activity of ICIs, alone or in combination with other immunotherapeutic agents (such as anti-cancer vaccines), is summarized.

### 3.5. Immunotherapy in mCRPC: Combining ICIs with ARTAs

Several studies are currently assessing the combination of ICIs with other agents for potentially synergistic effect, looking for a better efficacy of the PD-1/PD-L1 blockade in PCa. As for the combination of ARTAs and ICIs, enzalutamide showed promising results when co-administered with pembrolizumab in KEYNOTE-199 and KEYNOTE-365 trials, given the higher PD-L1 expression over dendritic cells’ plasmalemma displayed in mCRPC patients progressed on enzalutamide [63]. Meanwhile in KEYNOTE-199 the addition of pembrolizumab was demonstrated to improve the clinical activity of enzalutamide in chemotherapy-naive mCRPC men progressed on enzalutamide monotherapy [64], the interim analysis of the phase IB/II KEYNOTE-365 study pointed out an encouraging disease control rate (DCR) related to this drug’s combination in enzalutamide-naive patients who have failed abiraterone acetate [65]. Nowadays, enzalutamide and pembrolizumab are being tested among enzalutamide-naive patients in the advanced hormone-sensitive setting (KEYNOTE-991, NCT04191096) as well as in the metastatic castration-refractory setting (KEYNOTE-641, NCT03834493) (Table 3). On the other hand, the combination of atezolizumab plus enzalutamide was not as fortunate as the previous combination, based on the IMbassador 250 results [66].

### 3.6. Immunotherapy in mCRPC: Combining ICIs with Chemotherapy

The clinical activity of the combination of immunotherapy and chemotherapy in PCa has been evaluated because of the increased sensitivity to killing by CD8+ cytotoxic T-lymphocytes of taxane-refractory malignant cells. Notably, hopeful results (14% ORR, 33% PSA response rate, 57% DCR, with median duration of response 4.9 months) have been gained from the interim analysis of the KEYNOTE-365 cohort B, in which the combination of pembrolizumab and docetaxel was studied [67]. The CheckMate 9KD trial displayed interesting data on chemotherapy-naive patients with CRPC treated with nivolumab and docetaxel: ORR 40%, PSA response rate 46.9%, and median radiographic PFS 9.0 months [68]. Furthermore, the ongoing phase III KEYNOTE-921 trial (NCT03834506) is testing pembrolizumab with docetaxel for mCRPC after treatment with enzalutamide or abiraterone acetate, and the phase III CheckMate 7DX study (NCT04100018) is currently investigating the activity of nivolumab and docetaxel in mCRPC after enzalutamide and/or abiraterone acetate (Table 3). 

### 3.7. Immunotherapy in mCRPC: Other Immuno-Combinations

The rationale for using PARP inhibitors or vascular endothelial growth factor receptor (VEGFR) tyrosine kinase inhibitors in combination with ICIs to have a synergistic action in the PD-1/PD-L1 blockade is well established [69,70]. The co-administration of durvalumab and olaparib was evaluated in a phase II study among 17 patients with mCRPC progressing after enzalutamide and/or abiraterone acetate, pointing out a radiographic and/or PSA response of 53% [71]. Of note, about half of the studied population had germline or somatic alterations in their DDR genes. Currently, the KEYNOTE-365 cohort A is studying the combination of pembrolizumab with olaparib in mCRPC previously treated with docetaxel, and the phase IB/II JAVELIN PARP Medley trial (NCT03330405) is assessing avelumab used with talazoparib in locally advanced and metastatic solid tumors (including CRPC). Moreover, the above-mentioned phase II CheckMate 9KD study is including a nivolumab plus rucaparib arm for patients with mCRPC, while the phase III KEYLYNK-010 study (NCT03834519) is currently comparing the combination of pembrolizumab and olaparib with enzalutamide or abiraterone in taxane-refractory mCRPC men (Table 3). With regards to anti-VEGFR TKIs, the combination of cabozantinib with atezolizumab showed a promising clinical activity in the COSMIC-021 cohort 6, when studied in mCRPC patients progressed on at least one ARTA: ORR 32% (CR 4.5% and PR 27%) and DCR 80%, with a 12.6 months-long median follow up [72]. Consequently, this immune combination is today being compared with abiraterone or enzalutamide in the phase III CONTACT-02 trial (NCT04446117), in patients with mCRPC progressed on prior treatments with at least one novel hormonal agent (Table 3). Lastly, the antitumor activity of multiple ICIs-based regimens has been investigated also in prostate cancer, given the meaningful results obtained with other solid tumors (including renal cell carcinoma or melanoma). Notably, the combination of nivolumab and ipilimumab was shown to be particularly effective in AR-V7 expressing mCRPCs, with a 25% of ORR in patients with measurable disease. Furthermore, a trend towards increased ORR was highlighted in dMMR patients [73].

### 3.8. Immunotherapy in mCRPC: Where Are We Running?

In conclusion, the dMMR/MSI-H molecular phenotype is therapeutically relevant in prostate cancer, given the related meaningful and durable response to the PD-1/PD-L1 blockade. Moreover, the dMMR/MSI-H status may be somatically acquired during the disease evolution. Even though this phenotype characterizes a small portion of PCa patients, these findings bear out the need to screen all patients with metastatic PCa for dMMR/MSI-H in order to identify who can benefit the most from immunotherapy [50]. Circulating tumor DNA assays may also be considered as a future and feasible way to detect patients harboring *MMR* genes’ mutations [74]. Further studies are required to unveil the mechanisms of resistance in dMMR/MSI-H patients who do not respond to ICIs. At the same time, the novel combinatorial approaches could increase the immunotherapy sensitivity of all metastatic PCa patients, by combining agents with complementary anti-tumor activity in order to convert an immunosuppressive “cold tumor” to a responsive “hot tumor”.

Ongoing clinical trials investigating the immunotherapy approaches are reported in Table 2 and Table 3.

## 4. PI3K and Akt Pathway

### 4.1. Functioning and Role in Carcinogenesis Process

**Figure 4:**
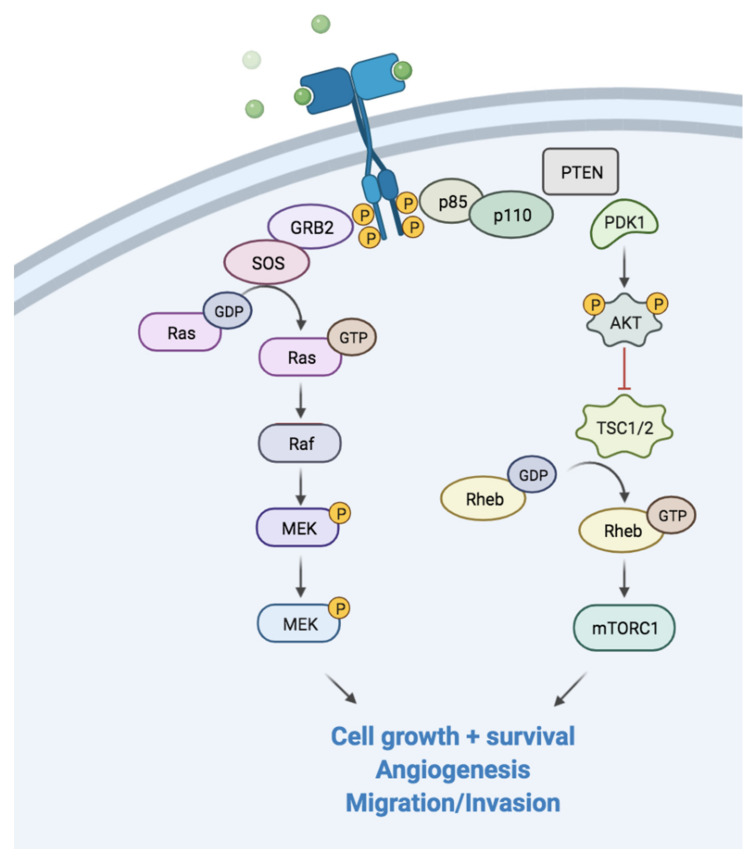
Schematic figure of the PI2K/Akt pathway. Following RTKs binding to the growth factors, the PI3K signaling pathway is activated. Akt: protein kinase B; GRB2: growth factor receptor-bound protein 2; PDK1: phosphoinositide-dependent kinase-1; PTEN: phosphatase and tensin homologue.

The oncosoppressor, *PTEN*, is a phosphatase acting mainly as an antagonist of the PI3K family [75]. The activation of the PI3K axis leads to the activation of protein kinase B (PkB or Akt) and the mammalian target of rapamycin (mTOR) signaling cascades, which have a key role in protein synthesis, cell growth, survival and migration. Moreover, *PTEN* intervenes in multiple cellular processes, such as senescence, apoptosis, extracellular microenvironment and both adaptive and innate immunity. This signaling pathway is also typically hyper-activated as a cellular response to stress, hypoxia and an unfavorable microenvironment with low pH or nutrients. The loss of *PTEN* causes an abnormal activation of the PI3K pathway, resulting in an uncontrolled stimulation of proliferation. Not surprisingly, this pathway is commonly altered in multiple cancers, such as prostate cancer, breast cancer and renal cell carcinoma [76,77] (Figure 4).

Mutations of the *PTEN* gene, that lead to the inactivation of PTEN, are quite frequent in prostate cancer: around 40% of localized prostate cancer and 70–80% of mCRPC present such alterations [12,78,79]. Loss of *PTEN* function can be caused by a variety of molecular modifications, including epigenetic silencing, post-transcriptional modification like microRNA and promoter methylation, and post-translational alteration [80]. Detection of *PTEN*-loss was initially mainly assessed by fluorescence in situ hybridization (FISH), but this technique may underestimate the frequency of this alteration considering that multiple genetic aberrations can lead to *PTEN* loss. Therefore, immunohistochemistry (IHC) seems to be more precise. More recently, NGS approaches have been under evaluation as an alternative method [81,82]. Interestingly, *PTEN* alteration seems to be present since the early phases of cancer evolution and is maintained from the hormone-sensitive phase to the castration-resistant status [83]. The role of *PTEN* loss in prostate cancer is also highlighted by the reciprocal feedback interaction between the androgen receptor (AR) and the PI3K/Akt pathways. Several preclinical studies in mice and humans showed that AR transcriptional production is reduced in *PTEN* deficient tumors. Additionally, the blockade of AR signaling increases the PI3K activation cascade, enabling prostate cancer cell survival. Therefore, increased function of the PI3K-Akt-mTOR is associated with the evolution to castration-resistance status [84,85,86,87].

As for the inhibition of the PI3K axis, it has been shown to reduce the RE-1 silencing transcription factor (REST) protein expression, leading to an increased neuroendocrine differentiation of cancer cells. This process is also enhanced by AR inhibition, so the blockade of both pathways can increase the incidence of the aforementioned evolution [88].

### 4.2. Development of PI3K/AKT Targeted Therapy in Prostate Cancer

Molecules targeting PI3K/Akt are being studied alone or in combination with other agents, in particular with abiraterone. Ipatasertib (GDC-0068) is an orally available, highly-selective pan-Akt (Akt1, Akt2 and Akt3) inhibitor. In vitro studies showed that inhibiting Akt significantly decreases the downward cascade of this pathway [89,90]. Several phase I trials evaluated ipatasertib alone or in combination with ARTAs and chemotherapy in multiple advanced solid cancer, showing a tolerable safety profile [91,92,93]. In a phase II trial, patients with pretreated mCRPC were randomly assigned to receive ipatasertib 200 mg, ipatasertib 400 mg or a placebo plus abiraterone 100 mg and prednisone 5 mg [94]. Co-primary endpoints were radiographic PFS in the PTEN-loss population and intention-to-treat population (ITT). The results supported the superiority of the combination of abiraterone and ipatasertib at a higher dose over the placebo, especially in patients with PTEN-loss. Patients with PTEN-loss tumors treated with ipatasertib 400 mg had a higher radiographic PFS (HR = 0.39; 90% CI, 0.22–0.70) compared with patients without PTEN-loss (HR = 0.84; 90% CI, 0.51–1.37). The most frequent adverse events linked to ipatasertib were diarrhea and asthenia, but the most frequent grade 3–4 events related to the experimental drug were hyperglycemia and rash. The positive results of this phase II trial led to a phase III trial in the earlier setting.

In the phase III randomized double-blinded IPATential 150 trial, 1101 patients with treatment-naive mCRPC were randomly assigned to receive abiraterone 1000 mg once daily and prednisone 5 mg twice daily plus ipatasertib 400 mg once daily or a placebo [95]. The co-primary endpoints were radiographic PFS both in the PTEN-loss-by-IHC population and in the ITT population. The combination of abiraterone and ipatasertib was resulted to be statistically significantly better than the control arm in the PTEN-loss population: radiographic PFS was 18.5 months in the ipatasertib plus abiraterone group and 16.5 months in the placebo plus abiraterone group (HR 0.77; 95% CI 0.61–0.98; *p* = 0.034; significant at α = 0.04). However, the co-primary endpoint in the ITT population was not met as the radiographic PFS was 19.2 months in the ipatasertib plus abiraterone group and 16.6 months in the placebo plus abiraterone group (HR 0.84; 95% CI 0.71–0.99; *p* = 0.043; not significant at α = 0.01) [95]. Secondary endpoints (radiological and PSA response rates and median time to PSA progression) appeared to be better in the experimental arm in both the PTEN-loss and ITT population. An exploratory biomarker analysis of patients with PTEN-loss or PIK3CA/Akt1/PTEN-altered tumors, detected by NGS, showed that this population presented improved outcomes with the combination of abiraterone and ipatasertib. The safety profile was consistent with previous trials of Akt-inhibitors. Ipatasertib is currently being studied in combination with different agents, such as darolutamide and ADT, in the neoadjuvant setting in high risk, prostate cancer with PTEN-loss (NCT04737109) and in the metastatic setting in combination with atezolizumab and docetaxel (NCT04404140), or rucaparib in a phase I study (NCT03840200).

Capivasertib (AZD5363) is another orally available, potent inhibitor of all Akt isoforms [96]. In a phase I study, capivasertib was evaluated in combination with enzalutamide in patients with mCRPC pretreated with abiraterone [97]. Capivasertib showed a tolerable profile and promising response in patients harboring different mutations in the PI3K/Akt/mTOR pathway. Similarly to ipatasertib, most frequent grade 3–4 adverse events were hyperglycemia and rash in 20–25% of patients. Capivasertib has also been studied in combination with docetaxel in mCRPC in the randomized, placebo-controlled phase II ProCAID study [98]. The primary objective of composite PFS (PSA progression or soft-tissue/bone/clinical progression) was not met (PFS 7.03 months in the capivasertib arm and 6.7 months in the placebo arm). Contrastingly, the secondary endpoint of OS was surprisingly higher in the experimental arm (31.15 months in the experimental arm versus 20.27 months in the control arm). Both the PFS and OS data were consistent irrespective of the PI3K/Akt/PTEN pathway activation status. Capivasertib is also currently under evaluation in the hormone-sensitive setting in combination with abiraterone in PTEN-deficient prostate cancer in an ongoing phase III trial (NCT04493853).

## 5. Conclusions

The expanding knowledge on the molecular alterations guiding prostate cancer progression and resistance to treatment is paving the way to novel therapeutic approaches in a disease still lacking tailored therapies in addition to the second-generation hormonal agents. As well as in other types of solid tumors, wide genome analyses are making it possible to individuate targetable pathways, thus opening the field to a precision medicine approach. DDR, Akt, and MSI are hopefully just the tip of a wider iceberg that could be tackled with targeted therapies. To overcome treatment resistance, a combination of therapies with different mechanisms of action are promising approaches. Future efforts should be made to further explore the molecular alterations carried by prostate cancer in the different stages of the disease and to individuate and validate predictive factors of response.

## Figures and Tables

**Table 1:** Main trials testing various PARPi in metastatic prostate cancer.

NCT (Acronym)	Phase	Number of Patients	Experimental Arm	Control Arm	Setting	Status
TOPARP-A (NCT01682772) [18]	II	16	Olaparib	-	mCRPC (previously treated with chemotherapy and novel anti-androgens)	Active, not recruiting
TOPARP-B (NCT01682772) [19]	II	711 (161 with DDR gene alterations)	Olaparib (400 or 300 mg)	-	mCRPC (at least one previous taxane-based line of therapy)	Active, not recruiting
PROfound (NCT02987543) [9]	III	245 in cohort A, 142 in cohort B	Olaparib	Abirateroneor Enzalutamide	mCRPC (previously treated with Abiraterone or Enzalutamide, taxane-based chemotherapy was permitted)	Active, not recruiting
TALAPRO-1 (NCT03148795) [21]	II	128	Talazoparib	-	mCRPC (previously treated with Abiraterone or Enzalutamide and at least one taxane-based chemotherapy)	Active, not recruiting
TALAPRO-2 (NCT03395197)	III	1150 (estimated)	Talazoparib + Enzalutamide	Placebo + Enzalutamide	mCRPC	Recruiting
TALAPRO-3 (NCT04821622)	III	550 (estimated)	Talazoparib + Enzalutamide	Placebo + Enzalutamide	mHSPC	Recruiting
GALAHAD (NCT02854436) [22]	II	165	Niraparib	-	mCRPC (androgen receptor-target treatment and taxanes)	Ongoing, not recruiting
MAGNITUDE (NCT03748641)	III	765	Niraparib + Abiraterone	Placebo + Abiraterone	mCRPC	Ongoing, not recruiting
TRITON2 (NCT02952534) [23]	II	277	Rucaparib	-	mCRPC (one or two previous novel anti-androgens and one taxane-based chemotherapy)	Completed

Abbreviations: mCRPC: metastatic castration-resistant prostate cancer; mHSPC: metastatic hormone-sensitive prostate cancer.

**Table 2:** An overview of the ongoing clinical trials assessing immune checkpoint inhibitors alone or in combination with other immunotherapeutic agents in advanced prostate cancer.

NCT (Acronym)	Phase	Number of Patients	Experimental Arm	Control Arm	Setting	Pharmaco- Dynamic	Status
NCT04382898 (PRO-MERIT)	I/II	80	W_pro1/W_pro1 + goserelin/W_pro1 + cemiplimab + goserelin	-	mCRPC	mRNA liposome complex of five Ags, NSAA and anti-PD-1	Recruiting
NCT02933255	I/II	29	Nivolumab + PROST-VAC	-	mCRPC	Anti-PD-1 and virus-based vaccine targeting PSA	Recruiting
NCT03493945	I/II	113	BN-Brachyury + M7824/ BN-Brachyury + ALT-803/ BN-Brachyury + ALT-803 + epacadostat	-	mCRPC	MVA cancer vaccine, anti-PD-1/TGF-beta (M7824), IL-14 agonist (ALT-803) and IDO-1 inhibitor (epacadostat)	Recruiting
NCT02985957	II	497	Nivolumab/ ipilimumab or ipilimumab	Cabazitaxel	mCRPC	Anti-PD-1 and anti-CTLA-4	Recruiting
NCT03570619 (IMPACT)	II	40	Nivolumab + ipilimumab	-	mCRPC (CDK12 mutations)	Anti-PD-1 and anti-CTLA-4	Recruiting
NCT04104893 (CHOMP)	II	30	Pembrolizumab	-	mCRPC (CDK12, MLH1, MSH2, MLH3, PMS1, MSH6, PMS2 mutations or MSI-H)	Anti-PD-1	Recruiting
NCT03040791 (ImmunoProst)	II	29	Nivolumab	-	mCRPC (BRCA1/2, ATM, PTEN, CHEK2, RAD51C, RAD51D, PALb12, MLH1, MSH2, MSH6, PMS2 mutations)	Anti-PD-1	Recruiting
NCT03570619	II	40	Nivolumab + ipilimumab	-	Advanced solid tumors with biallelic CDK12 loss	Anti-PD-1 + anti-CTLA-4	Recruiting
NCT00583024 (APP22)	II	66	AdPSA	-	mCRPC	PSA AdV vaccine	Active, not recruiting

Abbreviations: mCRPC: metastatic castration-resistant prostate cancer; Ags: antigens; AdV: adenovirus; PD-L1: programmed death ligand 1; PD-1: programmed death protein 1; TGF: tumor growth factor; IL: interleukin; PSA: prostate-specific antigen; CTLA-4: cytotoxic T-lymphocyte antigen 4; IDO-1: indoleamine 2,3-dioxygenase 1.

**Table 3:** Ongoing clinical trials evaluating various immuno-combination-based approaches in advanced prostate cancer.

NCT (Acronym)	Phase	Number of Patients	Experimental Arm	Control Arm	Setting	Pharmaco- Dynamic	Status
NCT03170960 (COSMIC-021)	I/II	1732	Atezolizumab + cabozantinib	-	mCRPC	Anti-PD-L1 and anti-VEGF and MET TKI	Recruiting
NCT02861573 (KEYNOTE- 365)	I/II	1000	Pembrolizumab + olaparib (cohort A)/docetaxel (cohort B)/enzalutamide (cohort C)/abiraterone (cohort D)/lenvatinib (cohorts E-F)/vibostolimab (cohort G)/CBDCA + etoposide (cohort H)	CBDCA + etoposide (only in cohort H’s arm 2)	mCRPC	Anti-PD-1 in combination with: PARPi, taxane, ARSIs, TKI.	Recruiting
NCT03673787	I/II	51	Atezolizumab + ipatasertib	-	mCRPC (PTEN loss)	Anti-PD-L1 and inhibitor of the serine/threonine protein kinase Akt	Recruiting
NCT03330405 (JAVELIN PARP Medley)	I/II	216	Avelumab + talazoparib	-	Locally advanced or metastatic solid tumors (including CRPC)	Anti-PD-L1 and PARPi	Active, not recruiting
NCT03658447 (PRINCE)	I/II	37	177Lu- PSMA + pembrolizumab	-	mCRPC	Conjugate of a PSMA ligand and a beta-emitting radioisotope Lu177 and anti-PD-1	Active, not recruiting
NCT04109729 (Rad2Nivo)	I/II	36	Radium-223 + nivolumab	-	mCRPC	Radio-isotope Rad223 and anti-PD-1	Recruiting
NCT01688492	I/II	57	Ipilimumab + abiraterone	-	mCRPC	Anti-CTLA-4 and ARSI	Active, not recruiting
NCT03409458	I/II	52	Avelumab + PT-112	-	Advanced solid tumors	Anti-PD-L1 and a platinum agent complexed to a pyrophosphatase ligand (PT-112)	Recruiting
NCT02740985	I	307	Durvalumab + AZD4635	-	Advanced solid tumors	Anti-PD-L1 and adenosine A2A receptor antagonist	Active, not recruiting
NCT03805594	I	30	177Lu-PSMA + pembrolizumab	-	mCRPC	Conjugate of a PSMA ligand and a beta-emitting radio-isotope Lu177 and anti-PD-1isotope Lu177 and anti-PD-1	Recruiting
NCT03549000	I	344	NZV930 alone or + PDR001/ NIR178/ both	-	mCRPC	Anti-CD73, anti-PD-1 (PDR001) and A2AR antagonist (NIR178)	Recruiting
NCT04159896	II	49	CEP-11981 + nivolumab	-	mCRPC	Pan-TKI with selectivity for VEGF-R/TIE2 and anti-PD-1	Recruiting
NCT03338790 (CheckMate 9KD)	II	330	Nivolumab + rucaparib/ docetaxel/ enzalutamide	-	mCRPC	Anti-PD-1 with PARPi or taxane or ARSI	Active, not recruiting
NCT01867333	II	57	PROST-VAC + enzalutamide	Enzalutamide	mCRPC	Virus-based vaccine and ARSI	Active, not recruiting
NCT04446117 (CONTACT- 02)	III	580	Atezolizumab + cabozantinib	Enzalutamide or abiraterone acetate	mCRPC	Anti-PD-L1 and anti-VEGF and MET TKI	Recruiting
NCT03834493 (KEYNOTE- 641)	III	1200	Pembrolizumab + enzalutamide	Placebo + enzalutamide	mCRPC	Anti-PD-1 and ARSI	Recruiting
NCT04191096 (KEYNOTE- 991)	III	1232	Pembrolizumab + enzalutamide	Placebo + enzalutamide	mHSPC	Anti-PD-1 and ARSI	Active, not recruiting
NCT03834506 (KEYNOTE- 921)	III	1000	Pembrolizumab + docetaxel	Placebo + docetaxel	mCRPC	Anti-PD-1 and taxane	Active, not recruiting
NCT03834519 (KEYLYNK- 010)	III	780	Pembrolizumab + olaparib	Abiraterone or enzalutamide	mCRPC	Anti-PD-1 and PARPi	Active, not recruiting
NCT04100018 (CheckMate 7DX)	III	984	Nivolumab + docetaxel	Placebo + docetaxel	mCRPC	Anti-PD-1 and taxane	Recruiting
NCT03879122 (PROSTRA- TEGY)	II/III	135	Docetaxel + nivolumab (arm 1)/docetaxel + ipilimumab → nivolumab (arm 2)	Docetaxel (arm 3)	mHSPC	Taxane, anti-PD-1 and anti-CTLA-4	Active, not recruiting

Abbreviations: mCRPC: metastatic castration-resistant prostate cancer; mHSPC: metastatic hormone-sensitive prostate cancer; CBDCA: carboplatin; PARPi: poly (ADP-ribose) polymerase inhibitor; TKI: tyrosine kinase inhibitor; VEGF: vascular endothelial growth factor; ARSI: androgen receptor signaling inhibitor; NSAA: non-steroidal androgen receptor antagonist; PD-L1: programmed death ligand 1; PD-1: programmed death protein 1; PSMA: prostate-specific membrane antigen; CTLA-4: cytotoxic T-lymphocyte antigen 4.
